# Evaluating the Differential Effects of Valproic Acid on Wharton’s Jelly Mesenchymal Stem Cells

**DOI:** 10.15171/apb.2019.059

**Published:** 2019-08-01

**Authors:** Homa Salami, Seyed Javad Mowal, Rasoul Moukhah, Zahra Hajebrahimi, Seyed Abdolhakim Hosseini, Houri Edalat

**Affiliations:** ^1^Department of Molecular Genetics, Faculty of Biological Sciences, Tarbiat Modares University, Tehran, Iran.; ^2^Quality assurance Department, Production and Research Complex, Pasteur Institute of Iran, Tehran, Iran.; ^3^Aerospace Research Institute, Ministry of Science, Research and Technology, Tehran, Iran.; ^4^Molecular Medicine Center, Hamadan University of Medical Sciences, Hamadan, Iran.; ^5^Human Genetics Research Center, Baqiyatallah University of Medical Sciences, Tehran, Iran.

**Keywords:** Valproic acid, Wharton’s jelly mesenchymal stem cells, Potency, Neural differentiation

## Abstract

***Purpose:*** The histone deacetylases (HDAC) inhibitor, valproic acid (VPA), is a common antiepileptic drug and is attractive for its broad range of therapeutic effects on many diseases. It has been employed as an inducer of pluripotency in some cultured cells. Conversely, VPA has also been employed as an inducer of in vitro differentiation in many other cells. Therefore, we employed WJMSCs as a cellular target to evaluate the differential effects of of VPA on potency state and differentiation level of Wharton’s Jelly mesenchymal stem cells (WJMSCs) in various concentrations and different culture mediums.

***Methods:*** The isolated WJMSCs were cultured in DMEM (MSC medium). According to previous protocols, WJMSCs were treated with 0, 0.5 and 1 mM VPA in MSC or embryonic stem cell (ESC) medium and 2 mM VPA in neural differentiation medium. Real-time polymerase chain reaction (PCR) and western blot analysis were performed for evaluating the expression of pluripotency markers. MTT and caspase assays were also performed on VPA-treated cells.

***Results:*** The expression of pluripotency markers and the viability of the WJMSCs – determined by MTT assay – were significantly increased after 0.5 mM VPA treatment in ESC medium. A 2 mM VPA treatment in neural differentiation medium significantly diminished the expression of pluripotency markers and the viability of WJMSCs.

***Conclusion:*** According to our results, both VPA concentration and the medium context can influence VPA effects on WJMSCs. The differential effects of VPA on WJMSCs can reflect its wide range of effects in the treatment of various diseases.

## Introduction

Some small molecules are capable of alteration of many essential cellular processes such as apoptosis, proliferation and differentiation. The anticonvulsant and antiepileptic drug, VPA, for example, can mainly exert these effects through epigenetic modifications such as direct and indirect inhibition of histone deacetylases (HDAC) and DNA methyl transferases, respectively. Due to its wide range of activities, this epigenetic drug has been employed to treat many diseases like neurological diseases, cancer, HIV infections, addiction, etc.^1^ It has been shown that histone deacetylase inhibitors are also capable to promote pluripotency induction.^2^

Pluripotent stem cells with an embryonic origin have the high capacity to differentiate into different cell types. However, their usage is hampered as a result of ethical consideration, and rejection problem due to immune responses following transplantation.^3^ On the other hand, induced pluripotent stem cells that were initially created by retroviral introduction of *Oct-4, Sox-2, Klf4 and c-myc* (often referred to as OSKM) do not have these ethical and rejection problems. Nonetheless, the aforesaid induced pluripotent stem cells have high tumorigenic potential mainly as a result of insertional mutagenesis of viral vectors.^4^ The fact that induction of pluripotency is the result of epigenetic modifications has led scientists to investigate small molecules such as VPA for generation of pluripotent stem cells. Therefore, the problem attributed to tumorigenic potential of viral vectors could somehow be circumvented by application of epigenetic modifiers such as VPA. Additionally, unlike stability of virally-induced pluripotent stem cells, epigenetically-induced pluripotent stem cells are reversible.^2^ On the other hand, the differentiation effects of VPA in various conditions have been demonstrated in several studies, as well.^5-7^ Therefore, the aim of the present work was to study the differential effects of VPA on the level of differentiation and the potency state of Wharton’s jelly mesenchymal stem cells (WJMSCs). Increased potency demonstrates an increased number of cell types that a stem cell can produce after differentiation. Unlike totipotent (such as dividing egg before blastocyst stage of human development) and pluripotent (such as embryonic stem cells from inner cell mass) stem cells,^8^ mesenchymal stem cells (MSCs) are considered as multipotent stem cells that possess many excellent advantages over other kinds of multipotent stem cells. They can be readily obtained for autologous grafts with no ethical or rejection problems and are able to differentiate into all three embryonic lineages of ectoderm, endoderm and mesoderm.^9^ MSCs can be extracted from adult (such as bone marrow) and even fetal (such as umbilical cord blood and Wharton’s jelly) tissues. Fetal sources of MSCs are younger and are thus robustly proliferated and differentiated relative to their adult counterparts. Fetal MSCs have also less immune-rejection problem relative to the adult MSCs.^10^ Therefore, we employed WJMSCs as these cells are acquired and propagated non-aggressively with no ethical or rejection problems. To our knowledge, this is the first study on the effects of VPA on potency state and differentiation level of WJMSCs.

## Materials and Methods

### Isolation and culture of mesenchymal stem cells from Wharton’s jelly

In this experimental study, MSCs from Wharton’s jelly were isolated as previously described by Pirjali et al in 2013.^11^ All patients (mothers) were ethically informed about this research by signing our form of consent, in advance. Under sterile conditions, Wharton’s jelly (n=6) was removed from the blood vessels and minced into small pieces of about 2-3 mm^3^ with the sterile scalpel. Wharton’s jelly pieces were cultured on T25 tissue culture plastic flasks in low-glucose DMEM media supplemented with 10% FBS, 1% antibiotic-antimycotic solution, and incubated in incubator with 5% CO2 at 37°C for 7 days. After 7 days, tissue explants were removed by replacing the medium. After 1 week, when cultures reached confluence (70% to 80%), adherent cells were washed with PBS and harvested with trypsin (0.05%) and EDTA and replated onto five flasks for further expansion as follows: 1- untreated cells (control group) that cultured in ESC medium; 2- cells cultured in MSC expansion medium with 0.5 mM VPA (Sigma-Aldrich) for 5 days; 3- cells cultured in MSC expansion medium with 1 mM VPA for 5 days 4- cells cultured in embryonic stem cell expansion medium (80% DMEM F12 supplemented with 0.1 mM MEM Non-Essential Amino Acids, 0.1mM b-mercaptoethanol, 100 U/mL penicillin, 100 µg/mL streptomycin, 20% Knockout serum and 2 mM L-glutamine) with 0.5 mM VPA for 5 days; 5- cells cultured in embryonic stem cell expansion medium with 1 mM VPA for 5 days. Cells were harvested and the expression of selected genes was analyzed by real-time polymerase chain reaction (PCR).^6^ Viability^7^ and apoptosis^7^ assays were performed in an additional group of: 6- cells cultured in neural differentiation medium.

### Flow cytometry

The following monoclonal antibodies were employed for immune-phenotyping studies: CD105, CD90, and CD166 (R&D Systems, Minneapolis, MN), CD54, CD45, CD106, HLA-DR (eBioscience, San Diego, CA), and CD34 (Dako, Glostrup, Denmark). Isotype control antibodies were purchased from eBioscience (San Diego, CA). 10^5^-10^6^ cells were employed for each experiment. First, cells were detached by trypsin and 3% rat serum was used for blocking non-specific binding sites before treatment with specific monoclonal and isotype control antibodies in 100 µL PBS-BSA 3% for 1 hour at 4°C in dark. The cells were then fixed by 1% paraformaldehyde and analyzed by using flow cytometry (Partec, Münster, Germany). Gating was performed for forward cells and non-specific attachments were put away based on isotype control antibodies.^12^

### Neural differentiation

Neural differentiation was performed as described previously applying a 3-step procedure. In the first step, the cells were preinduced in DMEM LG medium supplemented with 20% FBS and 10 ng/mL bFGF (Roche, Germany) for 24 hours. After that, a full-term differentiation induction step was performed in DMEM LG supplemented with 2% FBS, 2% DMSO, and 200 µM BHA for 5 hours. Finally, the previous medium was replaced with a long term-induction medium containing 2% FBS, 2% DMSO, 200 µM BHA, 25mM KCL 2 mM VPA, 10 µM Forskolin, and 5 µg/mL Insulin in DMEM LG.^6,7^ All following experiments were performed 6 hours after differentiation.

### Real-time quantitative PCR

The expression of selected genes was analyzed in all samples by real-time PCR.^6,7^ Total RNA was isolated from samples using TRIzol reagent (Invitrogen, UK) according to the manufacturer’s recommendations. The quality and quantity of extracted RNA were assessed by gel electrophoresis on the 1% agarose gel and spectrometry at 260 and 280 nm. Total RNAs were treated with RNase-free DNaseI (Fermentase, Lithuania) to remove any unwanted DNA contamination. Prime Script^TM^ RT reagent kit (Takara, Japan) was used for cDNA synthesis according to the manufacturer’s recommendations.

Quantitative real-time PCR was done using StepOnePlus Real-Time PCR (Applied Biosystems, USA) using SYBR Green Master mix (Ex Taq II) (Takara, Japan) with 5 µL master mix, 0.2 µM forward primer, 0.2 µM reverse primer, 0.04 µL ROX reference dye II, 1 µL cDNA Template, and dH2O to a final volume of 10 µL. PCR program was: initial denaturation at 95°C for 2 minutes; followed by 40 cycles of denaturation at 95°C for 5 seconds and annealing at 60°C for 30 seconds. Melt curves for all genes were obtained to check PCR reaction for presence of nonspecific products and confirm the specificity of reaction. Changes in the fold number were evaluated using the 2^-RΔΔCt^ method. The expression of Glyceraldehyde-3-phosphate dehydrogenase gene (GAPDH) of each sample was measured as a normalization control. Specific primers were designed using Allele ID 6.0 software and GenBank (http://www.ncbi.nlm.nih.gov) and submitted to BLAST search against human genome to ensure that the sequences were specific just for the gene of interest and synthesized by Macrogen (South Korea) as mentioned in Table 1. NT-2 cell line was employed as the positive control sample.

**Table 1:** Primers for quantitative real-time PCR

**Gene**	**Forward and Reverse primer (5′–3′)**	**Amplicon size (bp)**
GAPDH	F: GTGAACCATGAGAAGTATGACAAC	123
	R: CATGAGTCCTTCCACGATACC	
Oct4A	F: CTTCTCGCCCCCTCCAGGT	496
	R: AAATAGAACCCCCAGGGTGAGC	
c- Myc	F: CTCCTACGTTGCGGTCACAC	142
	R: CGGGTCGCAGATGAAACTCT	
Klf4	F: TGCTCCCATCTTTCTCCACG	91
	R: TCCTGCCAGCGGTTATTCG	
Sox2	F: AAGACTAGGACTGAGAGAAAGAAGAG	171
	R: AAGAGAGAGGCAAACTGGAATC	

### Western blot analysis

Cells were lysed in lysis buffer (50 mM Tris-HCl at pH 8.0, 150 mM NaCl, 2 mM EDTA and 0.1% NP-40) that contains a protease inhibitor cocktail (Roche). The same amount from total proteins were separated on 12% sodium dodecyl sulfate-polyacrylamide gel electrophoresis (SDS-PAGE) and then transferred to the polyvinylidene difluoride (PVDF) membranes. The blocked PVDF membranes (applying electrophoresis with 8% fat-free milk in Tris-buffered saline [TBS] containing 0.5% Tween-20 at 37°C for 60 minutes) were incubated with specific primary antibodies. Then, they were incubated with horseradish peroxidase-conjugated secondary antibodies: 1- mouse monoclonal anti-c-myc (9E10) (sc-40, Santa Cruz Biotechnology), 2- rabbit polyclonal anti-Klf4 (ab106629, Abcam) and 3- mouse monoclonal anti-β-actin (Sigma-Aldrich) as positive internal loading control. Then membranes were washed three times with TBS with Tween-20 (TBS-T) buffer and addition of TBS solution before chemiluminescence detection procedure.^13^

### MTT assay

3-(4,5-Dimethylthiazol-2-yl)-2,5-diphenyltetrazolium bromide (MTT) reagent (Sigma, USA) was employed for a colorimetric viability assay. Briefly, 20 µL of 10 mg/mL MTT was added to 200 µL of culture media plus MSCs. After a 4-hour incubation at 37°C and replacing the medium with 200 µL dimethyl sulfoxide to dissolve formazan crystals produced after MTT cleavage. After that, an ELIZA microplate reader (BioTech Company, USA) was used for measurement of absorbance at 570 nm wavelength.^7^

### Caspase activity assay

The activity of caspase-3 and caspase-7 was measured using Caspase-Glo 3/7 reagent (Promega) in quadruplicate samples. A luminometer (Berthold) was employed to measure the luminescence of all samples after a 1-hour incubation time at room temperature.^7^

### Statistical analysis

Experiments were run as duplicates or triplicates. GAPDH was employed as reference internal control gene. Reaction efficiencies of each primer pair was calculated using LinReg PCR, version 12.x software (AMC, Amsterdam, Netherlands). The relative average fold change of expression was measured as described previously. Statistical analysis of results was carried out using Statistical Program for Social Sciences (SPSS) 17.0 software (SPSS Inc., Chicago, IL) for group-wise comparison between cell groups following various treatments. Statistical significance was determined by one-way ANOVA and chi-square tests. *P *< 0.05 was considered significant.^14^

## Results and Discussion

### Isolation and characterization of MSCs from Wharton’s jelly

**Figure 1:**
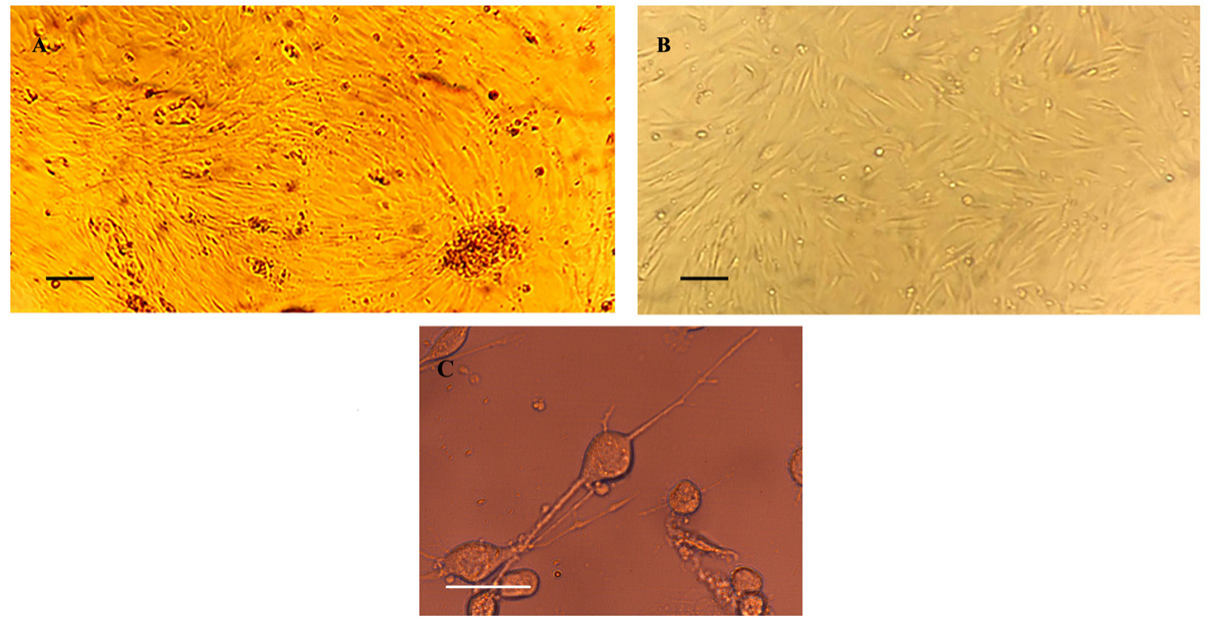
Phase-contrast microscopic illustration of the cells extracted from Wharton’s Jelly. (A) Attachment and proliferation of spindle-shaped cells occurs with an inside-to-outside direction after culturing the isolated tissue of Wharton’s Jelly. (B) The extracted WJMSCs in the second passage. (C) An illustrative figure of neural-like cells derived from WJMSCs at about 6 hours after initiation with neural induction medium. Figures of treated WJMSCs with VPA are not illustrated. (Scale bars: 20 µm).

The separated tissue pieces of Wharton’s jelly tissue start to attach to the plates with attached spindle-shaped cells spreading out of these fragments (Figure 1). Phenotypic appearance of cells did not change after VPA treatment.

Immuno-phenotyping by flow cytometry demonstrated that extracted cells were positive for CD105 and CD90 and negative for CD34, CD54, CD45, CD 106, CD166, and HLA-DR surface markers (see Figure S1, Supplementary file 1).

The human umbilical cord-derived MSCs have no ethical difficulties. These cells are considered as one of the most suitable sources in regenerative medicine that is mainly due to banking of umbilical cords (UC). MSCs do not have completely the pluripotency and self-renewal characteristics of real stem cells and undergo the process of aging after sequential divisions. However, they still retain their clinical benefits such as differentiation potential into chondrocytes, osteoblasts, myoblasts, adipocytes and neural-like cells.^12^

### Real time PCR and statistical analysis

Although embryonic stem cells have a wide variety range of differentiation potential, they remain ethical problems.^3^ Induced pluripotent stem cells however, have not these problems, because they are originally somatic cells that are induced into a pluripotent state through a reprogramming process by introduction of viral vectors containing the Yamanaka factors: c-Myc, Klf-4, Oct-4, and Sox-2 (all known as proto-oncogenes). But iPS cells have a great tumorigenic potential as a major obstacle, mainly due to the insertional activation of viral vectors and the usage of Yamanaka proto-oncogenes.^13^

Valproic acid (VPA, 2-propylpentanoic acid) was first introduced in 1962 as a drug for neuropsychiatric disorders such as epilepsy, depression, seizure and bipolar disease. The drug functions by increasing GABA neurotransmitter in the brain. This acidic compound is biochemically composed of an 8-C fatty acid molecule with little water solubility.^1^

VPA was first reported as an inhibitor of histone deacetylase (HDAC) in 2001 resulting in H3 and H4 hyper-acetylation.^1^ An obvious characteristic of pluripotent cells is an untightened chromatin network.^15^ Therefore, the aforesaid inhibitor of HDAC was proposed as a compound for reprogramming of somatic into pluripotent cells. The notion was lately confirmed in several experimental researches thus far by inducing the expression of pluripotency genes, i.e., Nanog, Nucleostemin, HoxB4, Bmi-1, ZFX and specifically, Oct4, Sox2, Klf4, and c-Myc.^2^

The aim of our study was to investigate the effects of VPA on human WJMSCs in various culture mediums of MSC, ESC, and neural differentiation. We estimated the expression of Oct4, Sox2, Klf4, and c-Myc and also viability and apoptosis assays of WJMSCs as an estimation for the level of potency or differentiation status of WJMSCs after such treatments.

The expression of c-Myc and Klf4 and viability of the WJMSCs were significantly improved after 0.5 mM VPA treatment in ESC medium (*P *< 0.05). A 2 mM VPA treatment in neural differentiation medium significantly lessened the expression of pluripotency markers and also viability of these cells (*P *< 0.05).

To our knowledge, this is the first report of evaluating the differential effects of VPA on WJMSCs and a previous study reported the effects of VPA on cord blood mesenchymal stromal cells (CBMSCs).^16^ Previous studies have shown that CBMSCs are known to be more effective in the process of scarless wound healing than WJMSCs.^17^

2^^-RΔΔCT^ formula was used to investigate the relative expression of the genes under study to GAPDH in WJMSCs with and without VPA treatments.^14^ Melt curve analysis was performed to investigate the specificity of the primers and LinReg PCR software was employed for calculating primer efficiencies. Sequencing procedure was performed to validate PCR products (data not shown).

**Figure 2:**
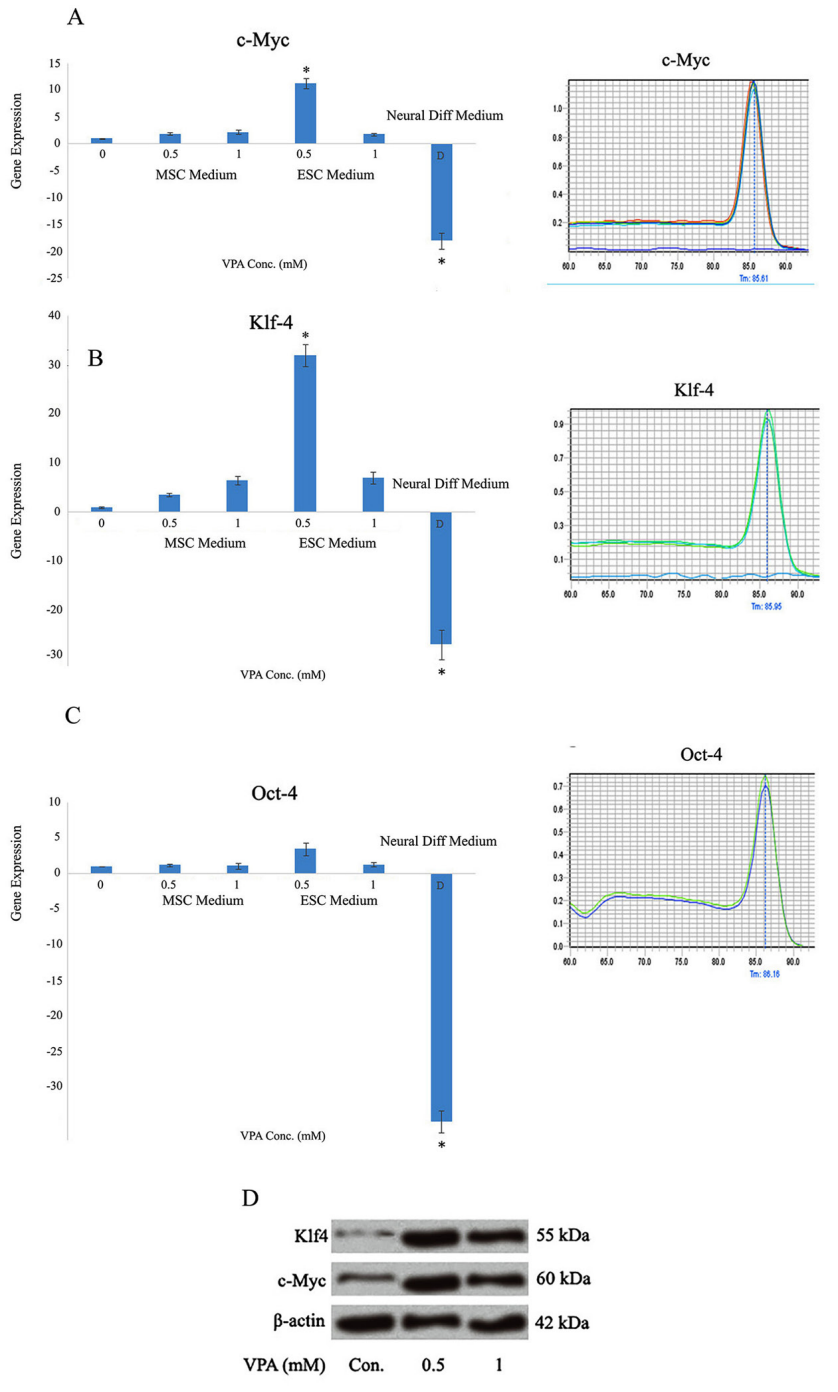
Representative melt curves (right panel) and gene expression patterns (left panel) for c-Myc (A), Klf4 (B), and Oct-4 (C) genes in MSCs extracted from umbilical cord cultured in MSC or ESC media in the presence or absence of VPA. The identical temperatures in each curve represent specificity of the primers. (A), (B) The expression of c-Myc and Klf4 was significantly elevated in WJMSCs cultured in ESC medium after a 0.5 mM VPA treatment and decreased in such cells treated with neural differentiation medium (containing 2 mM VPA concentration) compared with control (*P* < 0.05). No amplifications were detected in negative and no-RT controls. (C) Oct-4 was significantly reduced in neural differentiation media relative to the control cells (*P* < 0.05). (D) Western blot demonstrated increased level of Klf4 and c-Myc expression in ESC-cultured WJMSCs after VPA treatment compared to the control (with no VPA, *P* < 0.05).

Increased expression of c-Myc was observed in WJMSCs under all treatments (*P *< 0.05). The highest c-Myc expression was related to the cells cultured in ESC medium and under 0.5 mM VPA treatment related to control (MSCs cultured in ESC medium with no VPA treatment). c-Myc expression was significantly down-regulated in the cells cultured in neural differentiation medium using protocol of Woodbury relative to control sample (*P *< 0.05) (Figure 2A). Increased Klf4 expression was also observed for the cells under all treatments (*P *< 0.05). The highest Klf4 expression was also related to the cells cultured in ESC medium, under 0.5 mM VPA treatment, but the gene was significantly (*P *< 0.05) down-regulated in neural differentiation medium (Figure 2B). Oct-4 expression was not changed in pluripotency induction media, but there was a significant repression in the cells cultured in neural differentiation media (Figure 2C). No expression was observed for Sox-2 in WJMSCs either with or without treatments. No amplification was detected either in negative controls or no-RT samples and all replicate experiments revealed similar results. Western blot also indicated an increased level of Klf4 and c-Myc expression in ESC-cultured WJMSCs after VPA treatment relative to control (with no VPA treatment) (*P *< 0.05) (Figure 2D).

Collectively, we demonstrated an increased (*P *< 0.05) c-Myc and Klf-4 expression in WJMSCs and neither Oct-4 nor Sox-2 was shown to be expressed in these cells in this work. Even treatment of WJMSCs with combination of VPA and ESC medium did not change the results for Sox-2. The expression for Oct-4 was significantly (*P *< 0.05) recovered after such treatment, instead (with more than 10 times of elevation). Klf4 was also increased (*P *< 0.05) about 30 times after treatment. Although our previous study has demonstrated Oct-4 expression, no Sox-2 and HoxB-4 expression were detected in UVMSCs.^12^ The absence of Oct-4 expression in the current study might be due to the difference in the cell source that is Wharton’s jelly versus umbilical vein or it might be due to specific detection of transcript variant 1 of Oct-4 instead of identifying all transcript variants of the gene, in the current study. Additionally, our earlier study employed the immunocytochemistry method to evaluate Oct-4 protein, while we investigated Oct-4 expression at mRNA level.^12^ Another study however, demonstrated Oct-4 and Nanog expression in WJMSCs.^18^

Previous studies have shown that human first-trimester amniotic fluid stem cells express all four pluripotency markers (c-Myc, Klf-4, Oct-4 and Sox-2), although with lower levels of expression compared to ESCs. These cells can be induced to pluripotency by the means of 1 mM VPA alone without using any viral vectors.^19^ In contrast, although the mid-trimester amniotic fluid stem cells expressed Oct-4 and c-Myc in MSC culture medium, the expression of Nanog, Sox-2, and Klf-4 was not obvious enough to be detected. ESC culture however, made some changes in favor of reprogramming that were up-regulated expression of c-Myc and Oct-4 and the appearance of Sox-2 and Klf-4, but not Nanog. VPA treatment however, increased the expression of all the above-mentioned pluripotency markers also without using any viral vectors.^18^

On the other hand, we demonstrated that a higher VPA concentration in another medium context resulted in neural differentiation of the cells. Our finding is in accordance with other studies in which lower concentrations of VPA have also been demonstrated in inducing proliferation in a mouse embryonic mesenchymal cell line,^20^ while higher concentrations prevent proliferation of MSCs.^21^ Although lower concentrations (0.2 mM) of VPA were effective in serum-free proliferation and engraftment efficiency of CD45^+^34^+^ progenitor cells, a higher concentration was defined as an inhibiting factor of cell viability and was enough to induce apoptosis and differentiation, specifically in the presence of the serum.^22^ There are various reports on the beneficial effects of lower amounts of VPA pretreatment on chemotaxic, immunosuppressive, proangiogenesis, and self-renewal activities of implanted MSCs.^23^ Higher concentrations and longer duration time of exposures (>5mM for more than 3 hours) of VPA inhibited cellular proliferation.^16^ VPA effects on cellular proliferation can also be influenced by maturity of the cell. Immature cells are resistant to higher concentrations of VPA.^16^ Therefore, we infer this notion that a specific defined VPA concentration that induces reprogramming of mature differentiated cells is able to induce differentiation of immature cells.

Therefore, for the first time we propose that VPA has a tolerating effect on the cells to lead them towards homeostasis. For example, in cancer, the anti-cancer feature of VPA evolves, in development, the anti-differentiation feature and in cells with lower levels of potency, the pro-pluripotency feature of VPA would be revealed. It’s no surprise because many gene expression alterations in development and ageing occur quite inversely. As what is useful in development might be lethal in aging time^24-26^ and as a rule of thumb, epigenetic mechanisms play a pivotal role in differentiation and increased potency of the cells.

### Viability test and caspase assay

**Figure 3:**
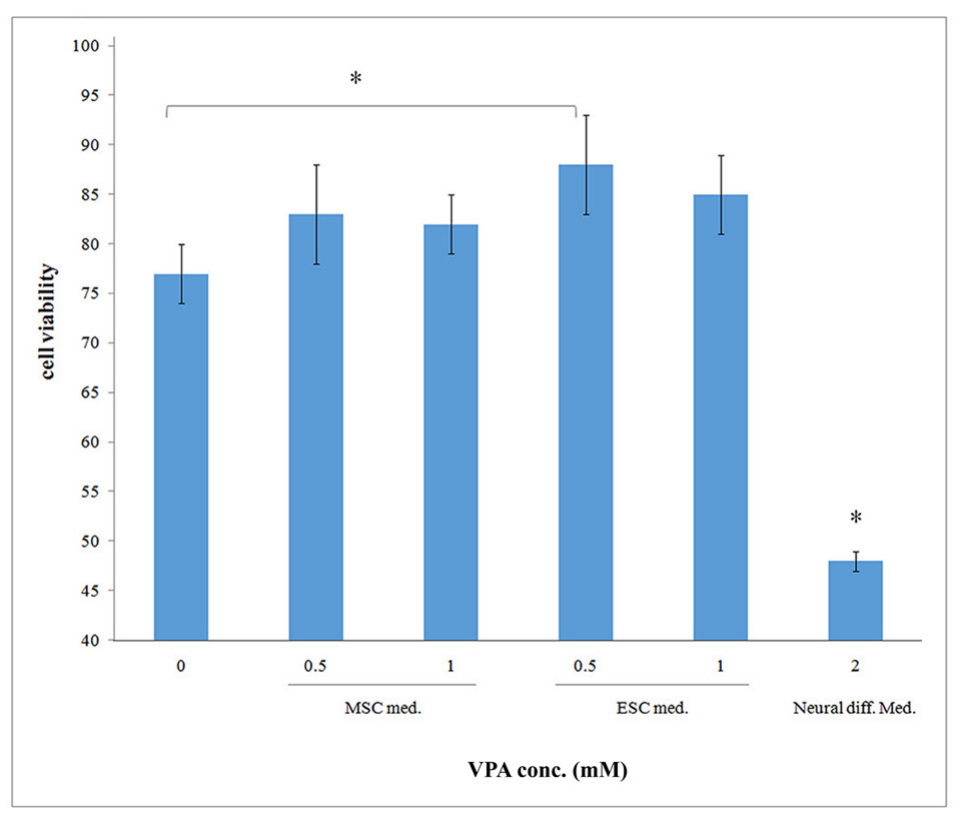
The highest viability ratio among non-transfected groups compared to the control was obtained for WJMSCs cultured in ESC medium after 5 days of 0.5 mM VPA treatment. Cell viability was significantly reduced in neural differentiation medium relative to all groups of treatment (*P* < 0.05).

The number of viable cells using MTT assay was significantly enhanced after treatment of cells with 0.5 mM VPA in ESC medium compared to control sample (*P *< 0.05). Cell viability was significantly reduced in 2 mM VPA concentration in the context of neural differentiation medium (*P *< 0.05) (Figure 3).

**Figure 4:**
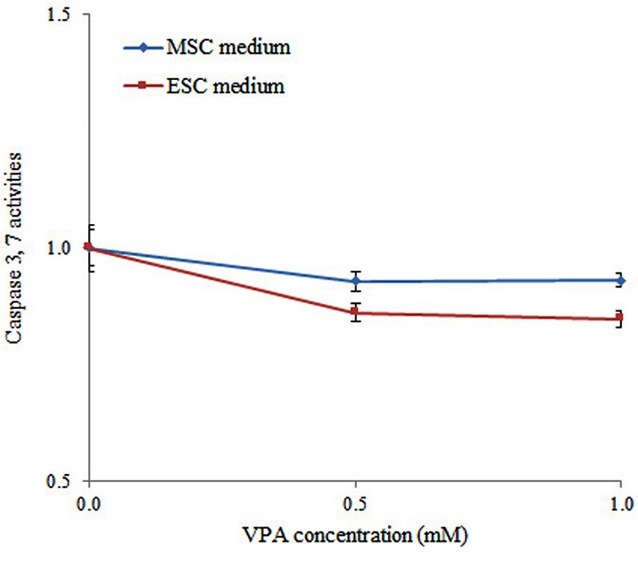
No significant apoptosis reduction was demonstrated between WJMSCs cultured in MSC or ESC medium following 0.5 mM or 1 mM treatments of VPA. Neither VPA concentration nor the background medium affected apoptosis in WJMSCs.

The activities of caspase-3 and caspase-7 were reduced in umbilical cord-derived MSCs cultured in ESC medium under 0.5 mM VPA treatment relative to control after treating with Promega Caspase-Glo 3/7 reagent (p>0.05). Non-transfected WJMSCs underwent a subtle degree of apoptosis reduction according to caspase 3, 7 activities in ESC culture relative to the MSC medium and no difference was demonstrated between 0.5 mM and 1 mM VPA concentrations. Briefly, no significant diminished apoptosis was indicated for all treatments in favor of enhanced potency (Figure 4).

Although a significant increase of survival was obtained after 0.5 mM VPA treatment in an ESC medium using MTT assay, no apoptosis difference was observed after such treatments. A similar observation was also obtained in which increased cell viability without significant apoptosis difference among various groups of treatment was reported.^27^ It is also possible that VPA can affect cellular death in various form of apoptosis, necrosis or autophagy and increase overall cytotoxicity. For example, a previous study also revealed the synergistic effects of melatonin and VPA on bladder cancer cells by increasing the expression of apoptosis, autophagy and necrotic genes.^28^ MTT is used to evaluate metabolic activity, cellular viability, proliferation and cytotoxicity after various treatments. Metabolic activity of the cells might be affected by some treatments without any alteration in cell viability or proliferation of the cells.^29^ For example, a recent interesting study has attributed the immunosuppressive capacity of transplanted MSCs to their metabolic activity. Enhancement of grafted MSC metabolic activity following VPA pre-treatment can further decline T-cell proliferation and increase immunosuppressive activity of MSCs.^30^ This finding is consistent with our MTT results in which enhanced metabolic activity of WJMSCs was obtained after VPA treatment without any change in the levels of apoptosis. On the other hand, our VPA treatments might not be enough to affect the levels of apoptosis in our study.

Finally, one of the main reasons for this inconsistency in our results might be the consequence of the background medium that has not been clearly mentioned before as an independent aspect. To our knowledge this is the first report of importance of background medium on VPA effect. A preliminary work emphasized the role of extracellular matrix on VPA effects that revealed an anti-proliferative and pro-differentiative properties of VPA in the presence of extracellular matrix.^20^

## Conclusion

We demonstrated VPA as a double-edge sword in different situations. We propose that VPA could have a tolerating effect on the cells to lead them towards homeostasis. As we obtained controversial results attributed to different VPA concentrations in various culture mediums, we propose that drug dosage should be obtained carefully to have the most therapeutic consequence and the least lethal effects. Finally, our experiments to somehow can pave the way for applying epigenetic modifiers such as VPA as a non-viral approach for increased potency induction.

## Ethical Issues

All the experimental procedures were approved by the medical ethics committee of Tarbiat Modares University.

## Conflict of Interest

The authors declare no conflict of interests.

## Acknowledgments

This research was financially supported by Iranian National Science Foundation (INSF)’s project no. 91053224.

## Supplementary files


Supplemenatry file 1 contains Figure S1.
