# Supplementary material for: Evaluating the Differential Effects of Valproic Acid on Wharton’s Jelly Mesenchymal Stem Cells
Source: Adv Pharm Bull. 2019 Aug 1;9(3):497–504. doi: 10.15171/apb.2019.059 (PMC6773934; doi:10.15171/apb.2019.059)
Supplement: Supplementary file 1 — Supplemenatry file 1 contains Figure S1. [file apb-9-497-s001.pdf]

Supplementary file 1

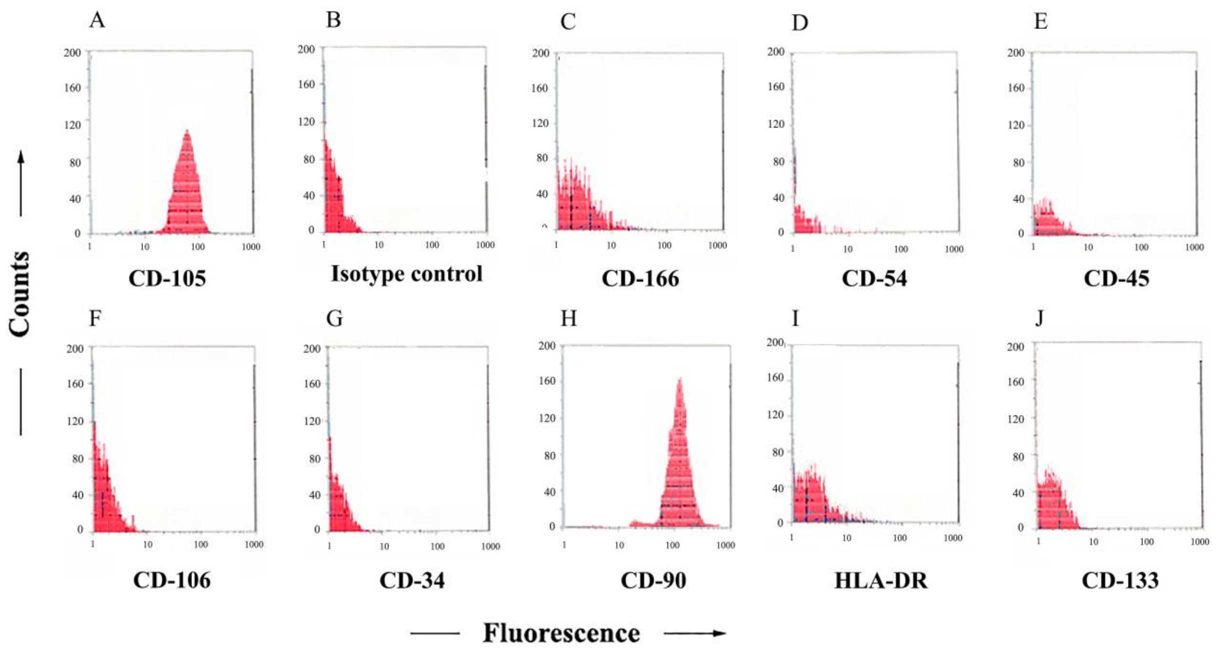

Figure S1. Immune-phenotyping studies of WJMSCs using flow cytometry by (A) CD-105, (C) CD-166, (D) CD-54, (E) CD-45, (F) CD-106, (G) CD-34, (H) CD-90, (I) HLA-DR, (J) CD-133 monoclonal antibodies. (B) Negative isotype-control antibodies were used.to eliminate the effect of non-specific antibodies. Cells were positive for CD-105 and CD-90, and negative for CD-166, CD-54, CD-45, CD-106, CD-34, and HLA-DR surface antigens.
